# A cost-effective RNA extraction and RT-qPCR approach to detect California serogroup viruses from pooled mosquito samples

**DOI:** 10.1038/s41598-024-52534-1

**Published:** 2024-01-29

**Authors:** Marc Avramov, Vanessa Gallo, Antonia Gross, David R. Lapen, Antoinette Ludwig, Catherine I. Cullingham

**Affiliations:** 1 Department of Biology, Carleton University, 1125 Colonel By Drive, Ottawa, ON K1S 5B6 Canada; 2 Ottawa Research and Development Centre, Agriculture and Agri-Food Canada, 960 Carling Avenue, Ottawa, ON K1A 0C6 Canada; 3 National Microbiology Laboratory Branch, Public Health Agency of Canada, 3200 Rue Sicotte, C.P. 5000, St. Hyacinthe, QC J2S 2M2 Canada

**Keywords:** Biological techniques, High-throughput screening

## Abstract

Mosquito-borne diseases pose ongoing global health concerns, demanding more cost-efficient methods to detect pathogens to support enhanced surveillance efforts. This study introduces an adapted TRIzol-based high-throughput RNA extraction protocol, tailored for the detection of California serogroup viruses in pooled mosquito samples in a rapid and cost-effective manner. This approach provided consistent RNA yields and sensitive viral detection relative to two commercial extraction kits (QIAGEN RNeasy Mini Kit and MACHEREY–NAGEL NucleoSpin RNA Plus Kit). The incorporation of a user-friendly and non-spiking-based RT-qPCR internal control designed for the 18S rRNA gene in mosquitoes minimizes potential false positives/negatives, improving the fidelity of viral detection outcomes. Effective RNA yields, purity, and successful target amplification across 25 mosquito species and varied pool sizes (1–50 mosquitoes per tube) affirm the reliability of our approach. The extraction method is cost-effective, with an incurred cost of $0.58 CAD per sample, in contrast to the $5.25 CAD cost per sample of the two kits, rendering it promising for mosquito-borne disease surveillance initiatives.

## Introduction

Vector-borne diseases transmitted by mosquitoes, ticks, or other arthropods present a global health challenge, contributing to over 700,000 deaths annually^1^. Climate and land use changes greatly influence pathogen range expansion^2–4^. Urbanization and international travel, for example, heighten transmission risks and the (re)introduction of mosquito-borne diseases (MBDs)^3,4^. In Canada, endemic pathogens like West Nile virus, eastern equine encephalitis virus, and California serogroup viruses (CSGv) are on the rise^3,5,6^. Some studies have demonstrated CSGv prevalence in mosquitoes, animals, and humans^5–8^, including a 2018–2019 MBD surveillance program in Southeastern Ontario, Canada, which identified several CSGv-positive mosquito pools. Despite this surge, CSGv cases are underreported, indicating a pressing need for enhanced surveillance and testing^3,9^. Surveillance initiatives can enhance detection and control, as evidenced by the SARS-CoV-2 epidemic^10^. With the expected increase of CSGv cases and potential exposure threats in North America, the demand for robust, sensitive, and specific surveillance and testing methods has intensified^3,6,11^.

Conventional RNA virus testing consists of sample processing, RNA extraction, followed by a PCR-based detection method. RNA extraction methods that depend on silica spin-column kits often suffer from supply limitations and cost constraints^10^. Addressing these challenges, Han et al.^10^ introduced a stable and accurate TRIzol-based, high-throughput and kit-free RNA extraction approach for SARS-CoV-2 detection. In this paper, we adapted the Han et al. method specifically for extracting RNA from pooled mosquito samples. Furthermore, to bolster the precision and reliability of viral detection outcomes, we developed a novel non-spiking-based and non-species-specific internal control designed for RT-qPCR. These efforts could significantly augment assessment of mosquito arboviral presence in any jurisdiction, especially those hampered by cost constraints associated with more routine testing procedures reliant on manufactured RNA extraction kits.

## Results and discussion

**Figure 1:**
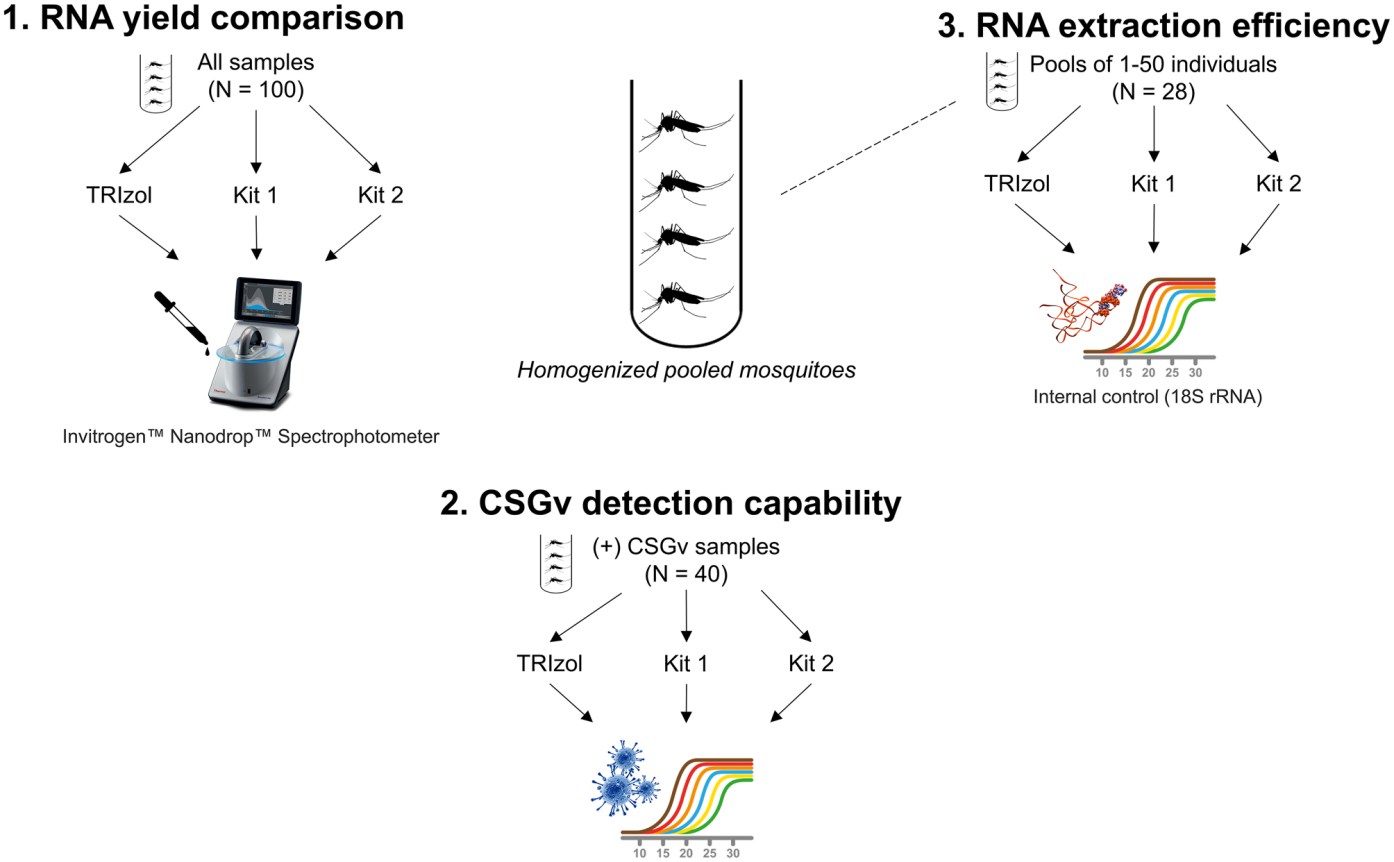
Schematic representation of the comprehensive comparative methodology. To compare the TRIzol method’s performance versus manufactured kits, the evaluation encompasses three key dimensions: (1) RNA yield quantification, (2) California serogroup virus detection capability, and (3) efficiency across various pool sizes. NOTE: “All samples” in (1) refers to the total samples that underwent RNA extraction, which includes the samples in (2), (3), and 32 additional samples used for negative controls or for internal control validation.

We used a comparative approach (see Fig. 1) to evaluate the performance of the TRIzol RNA extraction method versus two widely-used manufactured kits (QIAGEN™ RNeasy® and MACHEREY–NAGEL™ Nucleospin RNA Plus® kits, hereafter referred to as QIAGEN and MN). We used a total of 100 samples from the 2018–2019 eastern Ontario surveillance program (Environmental Change One Health Observatory Group), including 21 from the 2018 sampling season and 79 of the 2019 sampling season of which 19 and 21 were pre-screened positive for CSGv for 2018 and 2019, respectively. The first assessment involved extracting the same mosquito homogenates using all three extraction methods (N = 100) and measuring the differences in quantification of RNA yield obtained from each extraction technique. This step was crucial to ascertain if each method delivers consistent and sufficient RNA quantities for ensuing molecular analyses. Subsequently, using the CSGv screening results provided by the National Microbiology Laboratory (NML; Winnipeg, Manitoba, Canada), Canada’s reference diagnostic laboratory, as a benchmark, we used RT-qPCR to identify the presence of CSGv in known positive mosquito pools (N = 40) to confirm all methods provided dependable RNA extracts for the accurate detection of the target viruses. This test was necessary for gauging sample reliability during multiple freeze–thaw cycles during inter-lab transportation. Finally, the sensitivity of each extraction method across varying mosquito pool sizes (1–50 individuals) was examined (N = 28). This assessment sought to test the detection thresholds when only one or a few mosquitoes are available for testing. Each sample was tested in duplicates via RT-qPCR. Extraction and/or non-template controls were added at least once in each lane of the RT-qPCR plates for each run. The rest of the samples (N = 32) were used either as negative controls for the CSGv detectability RT-qPCR run or for the validation of the developed internal control.

In 2021, Han et al.^10^ introduced a scalable kit-free RNA extraction method, demonstrating its efficacy and specificity for SARS-CoV-2 detection in humans via liquid RNA extraction of swabbed tissue samples. This method requires the use of basic genomic laboratory equipment, such as a refrigerated plate centrifuge, compared to high-throughput extraction kits which often necessitate their own proprietary equipment. However, they highlighted the importance of validating and optimizing this approach for other infectious diseases. In this study, we focused on adapting this framework to target mosquito-borne arboviruses, specifically CSGv. The biggest challenge, as highlighted by Han et al.^10^, is the issue of phenol, chloroform, or salts contamination in phenol–chloroform based RNA extractions^12^, particularly in high-throughput applications. To address this issue, we employed meticulous pipetting by trained personnel to minimize the risk of phenol contamination before RNA precipitation with isopropanol, which was especially important for the phase-separation step. We omitted the use of glycogen contrary to Han et al.’s^10^ suggestion, as it consistently resulted in low A260/A230 ratios. The observed low RNA purity was possibly attributed to the presence of excess polysaccharides in the chitin-rich exoskeleton of mosquitoes^13^. The exclusion of glycogen was supported by successful RNA precipitation with isopropanol alone, obviating the need for glycogen in cell-rich samples like pooled mosquitoes. To enable broader downstream applications of the extracted RNA, including RNA sequencing and additional RT-qPCR diagnostics, we increased the final RNA volume to 30 µl, compared to Han et al.’s^10^ 10 µl. This modification was facilitated by consistent high RNA yields and successful validation of the internal control through RT-qPCR screening. By implementing these optimizations, our modified RNA extraction protocol offers reliable purity and yield, and broader application potential.

**Figure 2:**
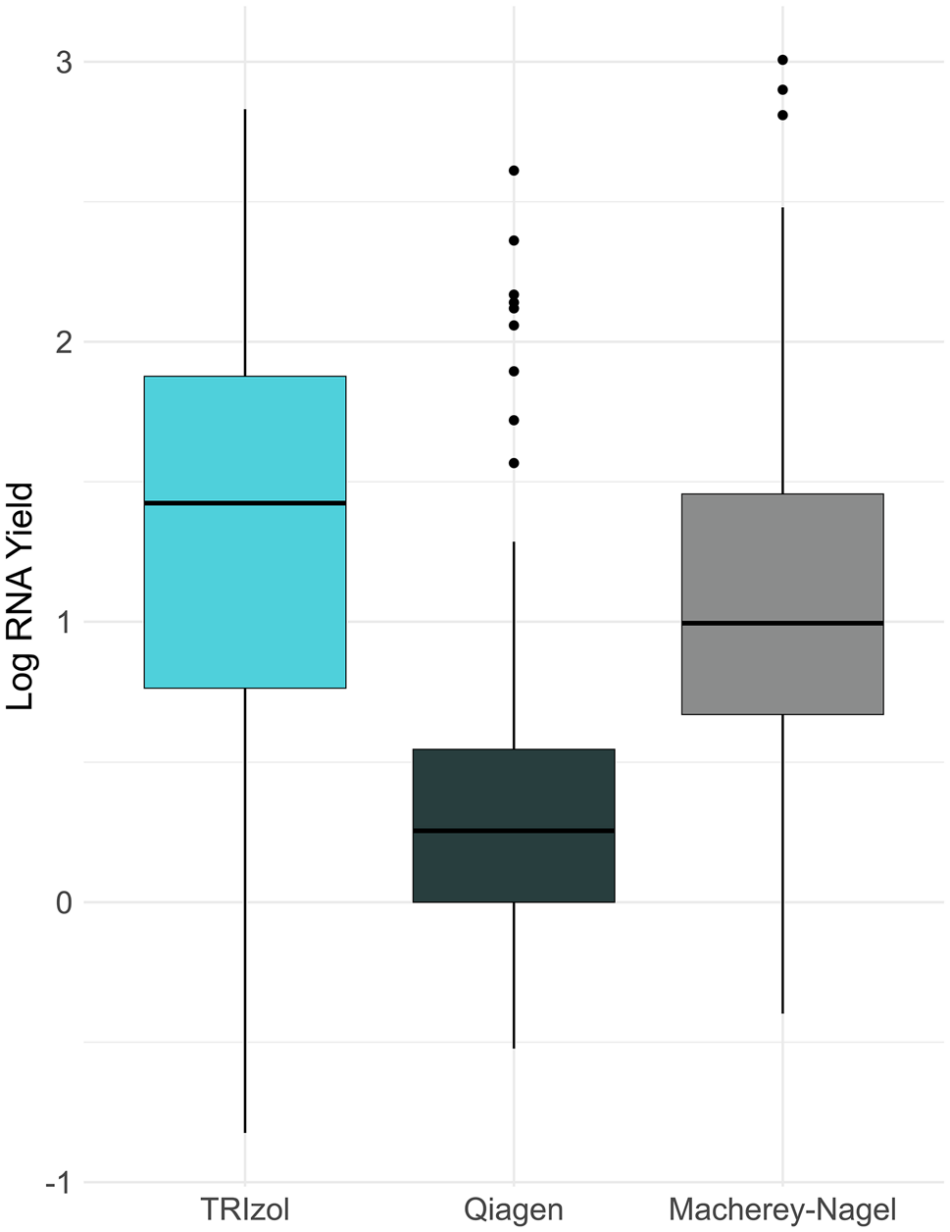
RNA yields of pooled mosquito samples (N = 100) for TRIzol and two commercial kits (QIAGEN RNeasy and MACHEREY–NAGEL Nucleospin RNA Plus). TRIzol extractions were done in a 96-well format while kit extractions were performed according to manufacturer protocols (with minor modifications). RNA concentration was quantified using NanoDrop One/OneC Spectrophotometer.

Phenol–chloroform-based RNA extraction methods often yield higher RNA quantities compared to kits due to their more aggressive nature in disrupting cells, non-selection for RNA size/species, and reduced loss of starting material^10,14^. In the analysis of 100 total sample extractions from each method, median yield for TRIzol was comparable to the kit methods (Fig. 2). The variability in yields across methods can be attributed to differences in mosquito abundance inherent to each sample. The range of yields observed for the TRIzol extracts demonstrated parity with those of QIAGEN and MN (Fig. 2, Supplementary Dataset S1). Notably, the utilization of a lower starting material volume for the TRIzol method (60 µl), in comparison to kit extractions (140 µl for QIAGEN; 200 µl for MN), did not compromise extraction efficiency while enabling a wider range of potential applications per sample.

**Figure 3:**
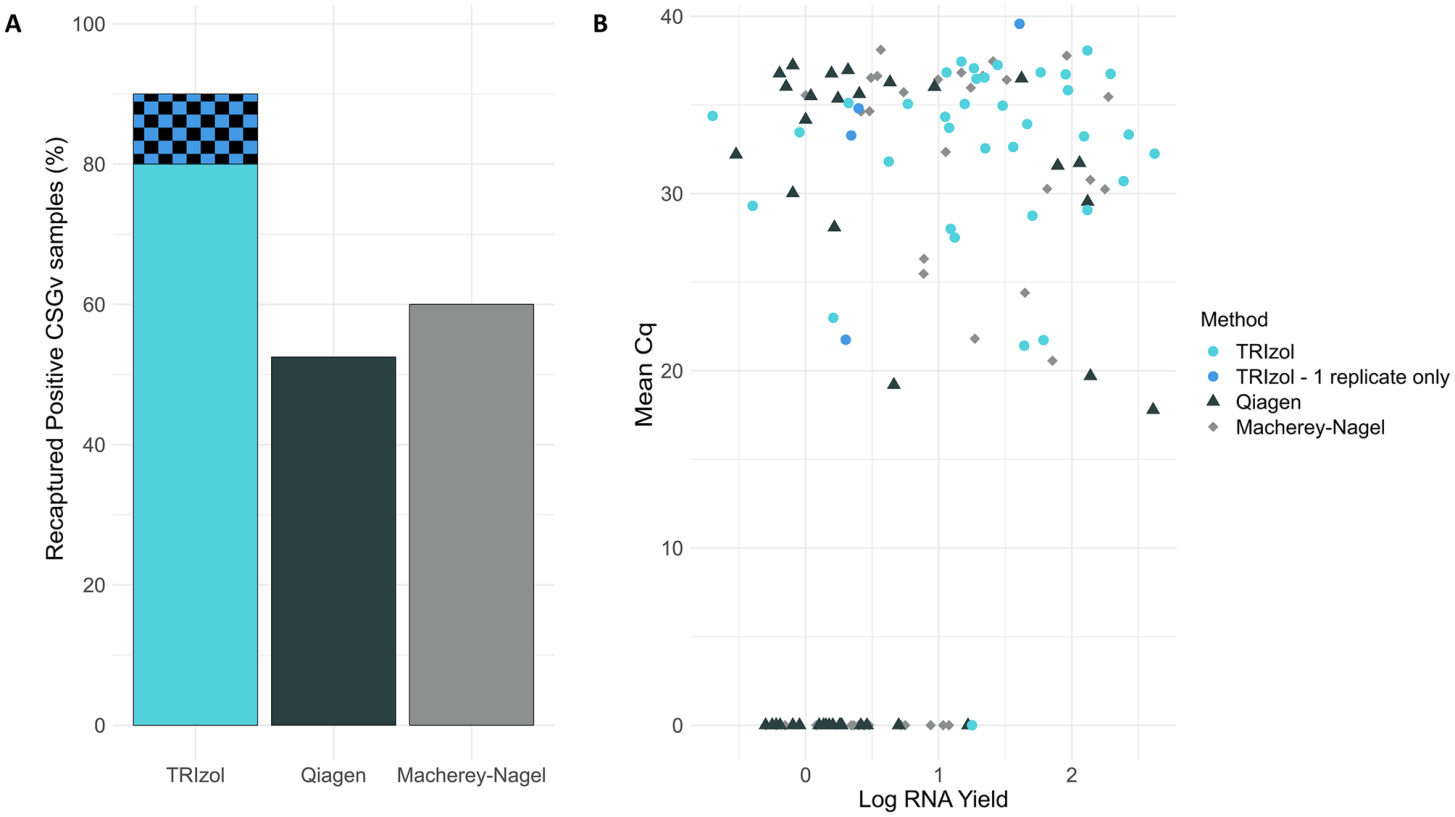
Reproducibility of California serogroup virus screening results via TRIzol, QIAGEN, and MACHEREY–NAGEL RNA extraction and subsequent RT-qPCR. Panel A depicts the percentage of samples that have been recaptured as positive for California serogroup viruses (CSGv) for each method. The checkered pattern indicates samples that were tested positive for CSGv in only one of the two TRIzol replicates and were not detected in either of the QIAGEN and MN replicates. Panel B shows that RNA yield (logged) and mean Cq values from the RT-qPCR were not correlated. Note that samples tested (N = 40) were initially screened positive by the National Microbiology Laboratory in Winnipeg, Manitoba, Canada and Carleton University, Ottawa, Ontario, Canada.

To evaluate viral detection performance, RNA was extracted from 40 mosquito homogenates previously confirmed as CSGv positive at the NML. Employing validated primer sets and probes^11^, the RT-qPCR analysis revealed that RNA extracts obtained using the TRIzol method successfully identified 32 out of the 40 (80%) samples previously characterized as CSGv positive in both replicates. Four samples positively detected CSGv in only one of the two TRIzol replicates, and these were not detected in either of the QIAGEN and MN replicates. This suggests possible sample degradation, undetectable viral loads, or false positives. The QIAGEN and MN extracts showed recapture rates of 52.5% and 60% in both replicates, respectively (Fig. 3a). Despite the anticipated challenges associated with multiple freeze/thaw cycles due to the samples being transported across Canada over the course of several years, the TRIzol method exhibited adequate sensitivity and performance. It is important to highlight that RNA yields did not show any correlation with quantification cycle (Cq) values, indicating that the methods extracted RNA non-specifically and that yields did not affect the detection capability of the targets across all three methods (Fig. 3b). This result further supports the reliability of our optimized TRIzol-based protocol in consistently detecting CSGv presence.

There appears to be limitations in accessible, user-friendly internal controls tailored for MBD screening via RT-qPCR. Many approaches rely on spiking-based internal controls^15,16^ which, while effective, increases complexity and elevated costs to high-throughput viral screening workflows. Our objective was to devise a dependable internal control that could integrate with multiplexed RT-qPCR assays through the straightforward addition of primers and a probe. Hoffman et al.^17^ previously assessed multiple primer pairs targeting various mosquito species to verify the integrity of RNA extracts. This validation aimed to ensure that the extracts were not degraded while testing for West Nile virus through PCR surveillance, thereby reducing the chances of false positives or negatives. Among these, the 18S417/18S920c primer set (23 nucleotides in length) demonstrated successful detection across 15 species from 8 genera (see^17^). This primer pairs amplicon’s identity was confirmed as the 18S rRNA gene of *Aedes albopictus*^17^. To integrate this control into the RT-qPCR assay, we developed a labeled probe to corroborate RNA integrity within each well, ensuring its unbiased detection across multiple species. Our assessments revealed that this primer pair and the associated probe were effectively detected in 25 species spanning 8 genera, all of which were representative of the sampling region (Table 1). Importantly, this internal control exhibited compatibility with the extracts obtained through all three methods (Table 1). The internal control’s robust detection rates suggest potential applicability to species beyond those directly determined in our assay. However, such extension would necessitate further validation procedures.

**Table 1:** Summary of internal control performance across mosquito species.

Species^a^	Mean Cq value (18S internal control)^b^		
	TRIzol	QIAGEN RNeasy	MN Nucleospin
*Aedes cinereus*	28.7	29.2	26.7
*Aedes vexans*	26.6	27.8	27.9
*Anopheles punctipennis*	22.9	25.7	20.6
*Anopheles quadrimaculatus*	25	35.5	19.3
*Anopheles walkeri*	24.5	24.1	20.3
*Coquillettidia perturbans*	25	24.2	23.4
*Culex pipiens-restuans*	29.1	26.7	25.8
*Culex restuans*	26	29.8	22.5
*Culex salinarius*	26	29.3	29.2
*Culiseta morsitans*	30	29	26.6
*Ochlerotatus abserratus-punctor*	25.1	26.1	28.1
*Ochlerotatus canadensis*	27.7	27.6	24.6
*Ochlerotatus communis*	20	28.9	19.4
*Ochlerotatus dorsalis*	31.7	22.6	22.3
*Ochlerotatus implicatus*	28.9	28.1	25.8
*Ochlerotatus intrudens*	29.3	31	25.7
*Ochlerotatus provocans*	27.2	29.8	27.8
*Ochlerotatus punctor*	27.1	33	26
*Ochlerotatus sticticus*	31.2	34.8	26.6
*Ochlerotatus stimulans*	22.3	22.2	24.9
*Ochlerotatus triseriatus*	26.6	25.1	23.7
*Ochlerotatus triseriatus-hendersoni*	22.7	23.1	21.5
*Ochlerotatus trivittatus*	36.3	26.2	30.6
*Psorophora ferox*	35.5	28.2	29.3
*Uranotaenia sapphirina*	34.4	28.5	20.6

^a^Encompasses 25 species sourced from 8 genera, all of which were representative of the Southeastern Ontario, Canada.

^b^Mean Cq value for two replicate RT-qPCR runs. See Supplementary Dataset S1 for replicate-specific Cq values.

**Figure 4:**
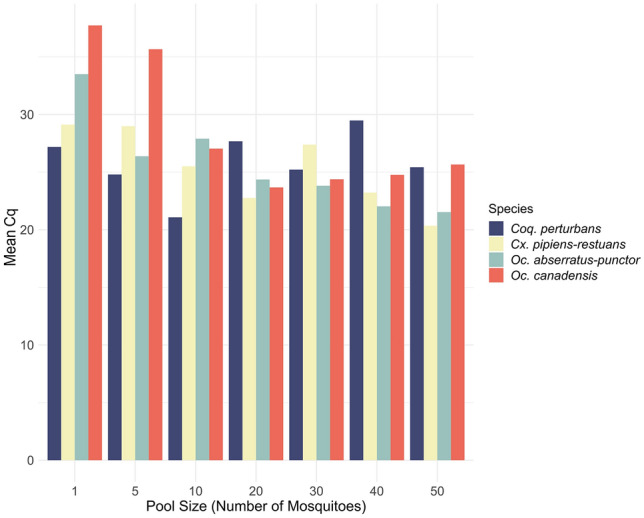
Internal control detection across varied pool sizes. TRIzol extracts outcomes of the internal control’s detection (mean Cq) across diverse pool sizes for RNA detection stability verification. Pool sizes ranged from one to fifty individuals. Four distinct mosquito species were tested to mitigate potential species-specific biases. The graph showcases the internal control’s consistent detection across a large spectrum of pool sizes.

We also verified RNA extraction efficiency across various mosquito pool sizes. The internal control’s detection capability was tested in pool sizes of one, five, 10, 20, 30, 40, and 50 individuals, using four different species to minimize potential species-specific biases linked to the 18S rRNA internal control gene targeting *Aedes albopictus*. Assay results show that the internal control was successfully detected in all pool sizes across the four species in TRIzol extracts (Fig. 4). This indicates that even small pool sizes, which could result from species rarity or seasonal factors during sampling, yield reliable viral surveillance outcomes. The method’s effectiveness extended to larger pool sizes as well, with no notable issues related to inhibition via potential DNA or protein carryover (Supplementary Dataset S1). The same trend was observed in the RT-qPCR results for QIAGEN and MN extracts, as detailed in Supplementary Dataset S1. Our findings emphasize the consistent performance of the developed method across a range of pool sizes and species, reaffirming the accuracy of our viral surveillance approach.

Time- and cost-effective frameworks are especially beneficial for large-scale MBD surveillance initiatives, where diagnostic labs often handle thousands of samples annually (depending on surveillance sampling duration). The time requirement for the entire screening process of our approach, including sample preparation, RNA extraction, and RT-qPCR, closely resembled Han et al.’s^10^ benchmark of 4 h for 96 samples, even when applied to mosquito pools. While our method aligns with kit procedures due to their single-channel pipette requirement, it is important to mention that the kit manufacturers offer 96-well RNA extraction formats for purchase, which would decrease the overall time requirement from 4 to 3 h. A notable advantage of our method is the substantial reduction in cost. Incorporating sample homogenization and RNA extraction, our approach effectively lowered the per-sample cost to $0.58 CAD (excluding instruments, and items comparable across methods including consumables, RT-qPCR detection, and technician expertise/time). In contrast, both QIAGEN and MN kits incurred a per-sample cost of $5.25 CAD, although opting for the kits’ 96-well format would reduce technician time by 0.75 times, which should be factored into overall cost considerations. Nonetheless, the TRIzol method’s ninefold cost reduction is particularly beneficial for resource-constrained laboratories or regions with heightened endemic pathogen presence. Also, the method’s compatibility with basic genomic laboratory equipment (see Methods) enhances its viability in such settings. While our manual pipetting approach proved efficient, it is important to caution that high repetitions of mixing via pipetting (see Methods) may cause repetitive straining injuries (RSI). Robotic pipetting instruments offer the potential for further automation and consistency, while minimizing the potential for RSI.

A few conceptual limitations are present in our study. Firstly, we recognize that the kits used for comparison are not optimized for liquid-based ento-virological RNA extractions, like the QIAGEN™ Viral Mini Kit. Hence, we can’t assert direct performance improvements of our method compared to all RNA extraction kits utilized in viral surveillance. Secondly, the modified homogenate volumes used for the two kits may not have been sufficient for total RNA extraction. Our rationale for adjusting the homogenate volume in both kits is twofold: firstly, to maintain the sample/buffer ratio within the manufacturer’s recommended tube capacity, and secondly, to ensure ample homogenate per sample for all extraction methods used in our study. Thirdly, we acknowledge that Cq values above 36, even after two replicates, may represent false positives attributed to probe breakdown and/or unspecific binding during RT-qPCR. However, CSGv values between 36 and 40 may also be attributed to potential low viral loads in small mosquito pool sizes. Finally, CSGv detection strategy was designed specifically for the detection of both snowshoe hare virus and Jamestown Canyon virus (see Methods). In usual screening protocols, all positive CSGv samples are tested again for these viruses independently. No documented cases for other CSGv (e.g. La Crosse virus) exist in our study region, thus we did not expect to capture other CSGv via RT-qPCR.

In conclusion, this study introduces a cost-effective high-throughput RNA extraction method tailored for mosquito arboviral surveillance, building upon the framework established by Han et al.^10^. The method’s proficiency in detecting mosquito-borne viruses in pooled samples underscores its significance in tackling vector-borne diseases that impose substantial global health burdens. Indeed, our RNA extraction and internal control strategy can be applicable to other mosquito-borne pathogens like West Nile virus and eastern equine encephalitis virus, as well as to various RNA species such as miRNA and siRNA. However, expanding these applications would require validation through separate and independent testing. Through careful protocol refinement, this TRIzol-based approach consistently demonstrated viable performance, not only in terms of RNA yield but also in cost-effectiveness. Our internal control strategy, which necessitates only primer and probe additions, addresses a critical gap in reliable MBD screening via RT-qPCR. Finally, the method’s resilience across diverse mosquito species and pool sizes and compatibility with basic laboratory setups further substantiate its applicability.

## Methods

### Samples

Mosquito sampling was conducted across multiple collection sites in 2018 and 2019 in eastern Ontario, Canada. Morphological manual mosquito species identification^18^ was carried out by the St-Hyacinthe laboratory of the Public Health Agency of Canada (St-Hyacinthe, Québec, Canada). Mosquitoes were combined in 1.5 ml Sarstedt tubes, grouped by species and according to specific collection site and date. The pooled tubes encompassed varying quantities of mosquitoes, ranging from 1 to 50 individuals. 2018 samples were first screened for CSGv via RT-qPCR at the NML (see^8^ for NML’s diagnostic framework). The mosquito homogenates were subsequently shipped to Carleton University for method development. The NML also processed and extracted RNA from the 2019 samples using an in-house adapted QIAGEN™ RNeasy® method (see^8^), but those extracts were screened for CSGv at Carleton University due to the SARS-CoV-2 diagnostics focus at the NML post-2020. All method development was done directly via the mosquito homogenate samples sent by the NML. All samples were processed using protocols that were approved by Carleton University’s Risk Assessment process^19^.

### Sample homogenization

Mosquito homogenization of all samples was done at the NML. One copper-clad BB bead and 1 ml of bovine albumin cell culture media [7.5% Bovine serum albumin fraction, 10 × M199 medium with Earl’s salts, 1 M Ultra-Pure Tris–HCl buffer (pH 7.5), Penicillin–Streptomycin (10,000 U/mL), ddH_2_O] was added to each Sarstedt tube containing mosquitoes. Samples were homogenized (QIAGEN™ Tissuelyser™ II) at 30 Hz for 2 min. Homogenates were centrifuged (Eppendorf™ 5810R) at 13,500 rpm for 4 min at 7 °C and stored at − 80 °C.

### Total RNA extraction using the RNeasy Mini Kit (QIAGEN™)

RNA extraction was performed using the RNeasy Mini Kit (QIAGEN™) according to the manufacturer’s instructions with minor modifications. The volumes for the sample homogenate, RLT buffer and 75% ethanol were modified to 140 µl, 280 µl and 280 µl, respectively. Centrifugation (Eppendorf™ 5430) steps were performed at 8000×*g* for 4 min instead of 1 min. The optional step of drying the membrane at 21,000×*g* for 1 min in a new collection tube was included. The RNA was solubilized in 30 µl of RNase-free water. RNA concentration was quantified using a NanoDrop™ One/OneC Spectrophotometer (Thermo Fisher Scientific™, USA).

### Total RNA extraction using the NucleoSpin RNA Plus Kit (MACHEREY–NAGEL™)

RNA extraction was performed using the NucleoSpin® RNA Plus Kit (MACHEREY–NAGEL™) according to the manufacturer’s instructions with minor modifications. The starting sample volume, lysis buffer (LBP) and binding solution (BS) were modified to 200 µl, 300 µl and 120 µl, respectively. Centrifugation (Eppendorf™ 5430) steps were performed at 11,000×*g* for 2 min instead of 30 s. The RNA was solubilized in 30 µl of RNase-free water. RNA concentration was quantified using a NanoDrop™ One/OneC Spectrophotometer (Thermo Fisher Scientific™, USA).

### Total RNA extraction using the TRIzol method

Han et al.’s^10^ protocol was adapted for large-scale RNA extraction of mosquito homogenates where 60 µl of mosquito homogenate was aliquoted into a 96-well PCR plate. A manual multi-channel pipette was used to add 140 µl of TRIzol™ Reagent (Invitrogen™ 15,596,018, USA) to each well. Samples were mixed by pipetting up-and-down 20 times and were incubated at room temperature for 10 min. 80 µl of chloroform was added into each well and samples were mixed by pipetting up-and-down 20 times. The plate was incubated at room temperature for 3 min and centrifuged (Eppendorf™ 5810R) at 3200 × g for 15 min at 4 °C. The clear upper aqueous layer of each well was transferred to a new plate and 80 µl of cold isopropanol (stored at − 20 °C) was added to each well. Samples were slowly mixed by pipetting up-and-down 20 times. The plate was incubated at room temperature for 10 min and centrifuged at 3200×*g* for 20 min at 4 °C. The supernatant was then discarded by inversion and the remaining RNA pellet was washed by adding 150 µl of cold 75% ethanol and mixing slowly by pipetting up-and-down 5 times. The plate was centrifuged at 3200×*g* for 10 min at 4 °C and the wash step was repeated. The RNA pellet was dried by incubating at 65 °C for 5 min on a heating block (Smart Block™, Eppendorf™, Germany). The RNA pellet was solubilized by adding 30 µl of DEPC-treated water (pre-warmed to 65 °C) and mixing slowly by pipetting up-and-down 20 times. The plate was incubated for 10 min at room temperature and RNA concentration was quantified using NanoDrop™ One/OneC Spectrophotometer.

### Real-time reverse transcription PCR (RT-qPCR)

A multiplex TaqMan™ RT-qPCR assay was used for the simultaneous detection of California serogroup viruses (CSGv), specifically Jamestown Canyon virus and snowshoe hare virus, alongside an internal control, targeting 18S ribosomal RNA. Specifically designed primers were utilized for both targets, with the CSGv primers from Wang et al.^11^ and the 18S primers from Hoffman et al.^17^. The 18S primers were used to serve as an internal control to confirm samples that do not contain virus are not false negatives. To assess this internal control as widely applicable, we validated its use across 25 mosquito species represented in our sample region. The probe for CSGv was labeled with the reporter dye 6-carboxyfluorescein (6-FAM) at the 5′ end and a minor groove binder (MGB) at the 3′ end, following the design by Wang et al.^11^. The internal control’s probe (sequence: 5′ – ATCAAGTGGAGGGCAAGTCTGGTG – 3′) was custom-designed using the PrimerQuest™ Tool (IDTech™, USA) labeled with the reporter dye 5-carboxytetramethylrhodamine (5-TAMRA) at the 5′ end and the quencher Iowa Black® RQ (IBRQ) (IDTech™, USA) at the 3’ end. The amplification reactions were carried out in a 20 µl reaction volume per well, comprising 4 µl of TaqMan™ Fast Virus 1-Step Master Mix (Thermo Fisher Scientific™ 4,444,432, USA), 5.25 µl DEPC-treated water (Thermo Fisher Scientific™ 4,387,937, USA), 4 µl of CSGv primers, 0.5 µl of CSGv probe, 1 µl of internal control primers, and 0.25 µl internal control probe. Before initiating the RT-qPCR run, the optical 96-well clear reaction plate (Applied Biosystems™ A36924, USA) was centrifuged at 950×*g* for 2 min (Eppendorf™ 5810R, Germany). The cycling conditions consisted of an initial incubation at 50 °C for 5 min followed by denaturation at 95 °C for 20 s for reverse-transcription. Subsequently, 45 amplification cycles were carried out, with denaturation at 95 °C for 3 s and annealing/extension at 60 °C for 30 s, using the QuantStudio™ 3 thermocycler (Thermo Fisher Scientific™, USA). The manufacturer-recommended detection thresholds of 0.04 (internal control) and 0.15 (CSGv) were employed to determine the presence of target RNA.

### Supplementary Information

Supplementary Information.

## Data Availability

All data supporting the findings of this study are available within the paper and its Supplementary Information.
