# Author Correction: A cost-effective RNA extraction and RT-qPCR approach to detect California serogroup viruses from pooled mosquito samples

**DOI:** 10.1038/s41598-024-69895-2

**Published:** 2024-08-22

**Authors:** Marc Avramov, Vanessa Gallo, Antonia Gross, David R. Lapen, Antoinette Ludwig, Catherine I. Cullingham

**Affiliations:** 1 Department of Biology, Carleton University, 1125 Colonel By Drive, Ottawa, ON K1S 5B6 Canada; 2 Ottawa Research and Development Centre, Agriculture and Agri-Food Canada, 960 Carling Avenue, Ottawa, ON K1A 0C6 Canada; 3 National Microbiology Laboratory Branch, Public Health Agency of Canada, 3200 Rue Sicotte, C.P. 5000, St. Hyacinthe, QC J2S 2M2 Canada

Correction to: *Scientific Reports* 10.1038/s41598-024-52534-1, published online 29 January 2024

The original version of this Article contained an error. In the Methods section, under the subheading ‘Real-time reverse transcription PCR (RT-qPCR)’, where an internal control probe for RT-qPCR was incorrect.

"The probe for CSGv was labeled with the reporter dye 6-carboxyfluorescein (6-FAM) at the 5′ end and a minor groove binder (MGB) at the 3′ end, following the design by Wang et al.^11^. The internal control’s probe (sequence: 5′ – CAGGTGCAAATGGA – 3′) was custom-designed using the PrimerQuest™ Tool (IDTech™, USA) labeled with the reporter dye 5-carboxytetramethylrhodamine (5-TAMRA) at the 5′ end and the quencher Iowa Black® RQ (IBRQ) (IDTech™, USA) at the 3′ end."

now reads:

"The probe for CSGv was labeled with the reporter dye 6-carboxyfluorescein (6-FAM) at the 5′ end and a minor groove binder (MGB) at the 3′ end, following the design by Wang et al.^11^. The internal control’s probe (sequence: 5′ - ATCAAGTGGAGGGCAAGTCTGGTG - 3′) was custom-designed using the PrimerQuest™ Tool (IDTech™, USA) labeled with the reporter dye 5-carboxytetramethylrhodamine (5-TAMRA) at the 5′ end and the quencher Iowa Black® RQ (IBRQ) (IDTech™, USA) at the 3′ end."

The original Article has been corrected.

